# Supplementary material for: Potential pathogens and antimicrobial resistance genes in household environments: a study of soil floors and cow dung in rural Bangladesh
Source: Appl Environ Microbiol. 2025 May 27;91(6):e00669-25. doi: 10.1128/aem.00669-25 (PMC12175521; doi:10.1128/aem.00669-25)
Supplement: Supplemental tables and figures — Tables S1 to S4 and Fig. S1 to S4. [file aem.00669-25-s0001.docx]

# Appendix to *Potential Pathogens and Antimicrobial Resistance Genes in Household Environments: A Study of Soil Floors and Cow Dung in Rural Bangladesh*

**Table S1. Cow-related characteristics of each household**

| Household id | Location during the day | Location at night | Visible cow dung in courtyard | Visible cow dung on floor | Use cow feces |
| 1 | Tied up outside | Free inside home with no barrier | Yes | No | Yes |
| 2 | Tied up outside | Free inside the mother’s home with no barrier | Yes | Yes | Yes |
| 3 | Tied up outside | In a different house within the compound | Yes | No | Yes |
| 4 | Tied up outside | In a different house within the compound | Yes | No | No |
| 5 | Tied up outside | Tied up inside the mother’s home with barrier | Yes | No | No |
| 6 | Tied up outside | Tied up outside | Yes | Yes | No |
| 7 | Tied up outside | In a different house within the compound | No | No | No |
| 8 | In a different house within the compound | In a different house within the compound | Yes | No | No |
| 9 | In a different house within the compound | In a different house within the compound | No | No | Yes |
| 10 | In a different house within the compound | Tied up outside | Yes | No | No |

**Table S2. Raw and processed read counts for each sample**

| Sample Type | Sample ID | Raw Reads (millions) | Processed Reads (millions) | Percent Retained (%) | Number of contigs |
| --- | --- | --- | --- | --- | --- |
| Cow | C1 | 16.2 | 10.817 | 66.8 | 208,094 |
|  | C2 | 19.111 | 12.992 | 68.0 | 227,540 |
|  | C3 | 14.247 | 9.641 | 67.7 | 213,280 |
|  | C4 | 7.18 | 4.896 | 68.2 | 206,038 |
|  | C5 | 7.457 | 4.938 | 66.2 | 186,040 |
|  | C6 | 17.057 | 11.401 | 66.8 | 226,440 |
|  | C7 | 9.699 | 6.647 | 68.5 | 187,853 |
|  | C8 | 17.606 | 11.695 | 66.4 | 210,926 |
|  | C9 | 15.997 | 10.919 | 68.3 | 204,773 |
|  | C10 | 28.384 | 12.330 | 43.4 | 203,794 |
| Soil | S1 | 1.372 | 0.789 | 57.5 | 76,858 |
|  | S2 | 2.096 | 1.184 | 56.5 | 122,737 |
|  | S3 | 0.588 | 0.259 | 44.1 | 37,002 |
|  | S4 | 2.627 | 1.432 | 54.5 | 86,007 |
|  | S5 | 1.445 | 0.767 | 53.1 | 100,592 |
|  | S6 | 10.449 | 5.276 | 50.5 | 237,927 |
|  | S7 | 1.093 | 0.516 | 47.2 | 58,302 |
|  | S8 | 1.834 | 1.085 | 59.2 | 105,734 |
|  | S9 | 0.769 | 0.392 | 51.0 | 50,360 |
|  | S10 | 11.008 | 6.028 | 54.8 | 193,209 |

**Table S3. All potential pathogen species detected**

| Genus | Species | Present in cow dung | Present in soil floor |
| --- | --- | --- | --- |
| *Abiotrophia* | *Abiotrophia defectiva* | 1 | 1 |
| *Achromobacter* | *Achromobacter xylosoxidans* | 1 | 1 |
| *Acinetobacter* | *Acinetobacter baumannii* | 1 | 1 |
| *Actinomadura* | *Actinomadura madurae* | 1 | 1 |
| *Actinomyces* | *Actinomyces israelii* | 1 | 1 |
| *Actinotignum* | *Actinotignum schaalii* | 1 | 1 |
| *Aerococcus* | *Aerococcus sanguinicola* | 1 | 1 |
| *Aerococcus* | *Aerococcus urinae* | 1 | 0 |
| *Aeromonas* | *Aeromonas caviae* | 1 | 1 |
| *Aeromonas* | *Aeromonas hydrophila* | 1 | 1 |
| *Aeromonas* | *Aeromonas veronii* | 1 | 1 |
| *Aggregatibacter* | *Aggregatibacter actinomycetemcomitans* | 1 | 1 |
| *Aggregatibacter* | *Aggregatibacter aphrophilus* | 1 | 1 |
| *Agrobacterium* | *Agrobacterium tumefaciens* | 1 | 1 |
| *Aliarcobacter* | *Aliarcobacter butzleri* | 1 | 1 |
| *Anaerococcus* | *Anaerococcus prevotii* | 1 | 0 |
| *Anaplasma* | *Anaplasma phagocytophilum* | 1 | 0 |
| *Arcanobacterium* | *Arcanobacterium haemolyticum* | 1 | 0 |
| *Aspergillus* | *Aspergillus flavus* | 1 | 1 |
| *Aspergillus* | *Aspergillus fumigatus* | 1 | 1 |
| *Aspergillus* | *Aspergillus nidulans* | 1 | 1 |
| *Bacillus* | *Bacillus anthracis* | 0 | 1 |
| *Bacillus* | *Bacillus cereus* | 1 | 1 |
| *Bartonella* | *Bartonella bacilliformis* | 1 | 0 |
| *Bartonella* | *Bartonella clarridgeiae* | 1 | 1 |
| *Bartonella* | *Bartonella elizabethae* | 1 | 0 |
| *Bartonella* | *Bartonella grahamii* | 1 | 0 |
| *Bartonella* | *Bartonella henselae* | 1 | 1 |
| *Bartonella* | *Bartonella quintana* | 1 | 1 |
| *Bordetella* | *Bordetella bronchiseptica* | 1 | 1 |
| *Bordetella* | *Bordetella parapertussis* | 1 | 1 |
| *Borrelia* | *Borrelia hermsii* | 1 | 0 |
| *Borrelia* | *Borrelia miyamotoi* | 1 | 0 |
| *Borrelia* | *Borrelia parkeri* | 1 | 1 |
| *Borrelia* | *Borrelia recurrentis* | 1 | 1 |
| *Borreliella* | *Borreliella burgdorferi* | 1 | 0 |
| *Borreliella* | *Borreliella mayonii* | 1 | 0 |
| *Brachyspira* | *Brachyspira hyodysenteriae* | 1 | 1 |
| *Brevibacillus* | *Brevibacillus brevis* | 1 | 1 |
| *Brucella* | *Brucella abortus* | 1 | 1 |
| *Brucella* | *Brucella melitensis* | 1 | 1 |
| *Brucella* | *Brucella suis* | 0 | 1 |
| *Burkholderia* | *Burkholderia cenocepacia* | 1 | 1 |
| *Burkholderia* | *Burkholderia cepacia* | 1 | 1 |
| *Burkholderia* | *Burkholderia mallei* | 0 | 1 |
| *Burkholderia* | *Burkholderia pseudomallei* | 1 | 1 |
| *Campylobacter* | *Campylobacter coli* | 1 | 1 |
| *Campylobacter* | *Campylobacter curvus* | 1 | 1 |
| *Campylobacter* | *Campylobacter fetus* | 1 | 1 |
| *Campylobacter* | *Campylobacter jejuni* | 1 | 1 |
| *Campylobacter* | *Campylobacter lari* | 1 | 1 |
| *Candida* | *Candida albicans* | 1 | 1 |
| *Candida* | *Candida auris* | 1 | 1 |
| *Candida* | *Candida dubliniensis* | 1 | 0 |
| *Cardiobacterium* | *Cardiobacterium hominis* | 1 | 1 |
| *Chlamydia* | *Chlamydia pneumoniae* | 1 | 1 |
| *Citrobacter* | *Citrobacter freundii* | 1 | 1 |
| *Clostridioides* | *Clostridioides difficile* | 1 | 1 |
| *Clostridium* | *Clostridium botulinum* | 1 | 1 |
| *Clostridium* | *Clostridium perfringens* | 1 | 1 |
| *Clostridium* | *Clostridium septicum* | 1 | 1 |
| *Clostridium* | *Clostridium tetani* | 1 | 1 |
| *Corynebacterium* | *Corynebacterium diphtheriae* | 1 | 1 |
| *Corynebacterium* | *Corynebacterium minutissimum* | 1 | 1 |
| *Corynebacterium* | *Corynebacterium striatum* | 1 | 1 |
| *Corynebacterium* | *Corynebacterium ulcerans* | 1 | 0 |
| *Coxiella* | *Coxiella burnetii* | 1 | 1 |
| *Cronobacter* | *Cronobacter sakazakii* | 1 | 1 |
| *Cryptococcus* | *Cryptococcus gattii* | 1 | 1 |
| *Cryptococcus* | *Cryptococcus neoformans* | 1 | 1 |
| *Edwardsiella* | *Edwardsiella tarda* | 1 | 1 |
| *Ehrlichia* | *Ehrlichia chaffeensis* | 1 | 1 |
| *Ehrlichia* | *Ehrlichia muris* | 1 | 0 |
| *Eikenella* | *Eikenella corrodens* | 1 | 1 |
| *Elizabethkingia* | *Elizabethkingia anophelis* | 1 | 1 |
| *Elizabethkingia* | *Elizabethkingia meningoseptica* | 1 | 1 |
| *Encephalitozoon* | *Encephalitozoon hellem* | 1 | 0 |
| *Encephalitozoon* | *Encephalitozoon intestinalis* | 1 | 0 |
| *Enterobacter* | *Enterobacter cloacae* | 1 | 1 |
| *Enterococcus* | *Enterococcus avium* | 1 | 1 |
| *Enterococcus* | *Enterococcus faecalis* | 1 | 1 |
| *Enterococcus* | *Enterococcus faecium* | 1 | 1 |
| *Enterococcus* | *Enterococcus gallinarum* | 1 | 1 |
| *Erysipelothrix* | *Erysipelothrix rhusiopathiae* | 1 | 1 |
| *Escherichia* | *Escherichia coli* | 1 | 1 |
| *Finegoldia* | *Finegoldia magna* | 1 | 1 |
| *Francisella* | *Francisella tularensis* | 1 | 1 |
| *Fusarium* | *Fusarium fujikuroi* | 1 | 1 |
| *Fusarium* | *Fusarium oxysporum* | 1 | 1 |
| *Fusobacterium* | *Fusobacterium necrophorum* | 1 | 1 |
| *Fusobacterium* | *Fusobacterium nucleatum* | 1 | 0 |
| *Gardnerella* | *Gardnerella vaginalis* | 1 | 1 |
| *Haemophilus* | *Haemophilus ducreyi* | 1 | 1 |
| *Haemophilus* | *Haemophilus influenzae* | 1 | 1 |
| *Haemophilus* | *Haemophilus parainfluenzae* | 1 | 1 |
| *Helicobacter* | *Helicobacter pylori* | 1 | 1 |
| *Kingella* | *Kingella denitrificans* | 1 | 1 |
| *Kingella* | *Kingella kingae* | 1 | 1 |
| *Klebsiella* | *Klebsiella aerogenes* | 1 | 1 |
| *Klebsiella* | *Klebsiella oxytoca* | 1 | 1 |
| *Klebsiella* | *Klebsiella pneumoniae* | 1 | 1 |
| *Legionella* | *Legionella micdadei* | 1 | 1 |
| *Legionella* | *Legionella pneumophila* | 1 | 1 |
| *Leptospira* | *Leptospira borgpetersenii* | 1 | 0 |
| *Leptospira* | *Leptospira interrogans* | 1 | 1 |
| *Leptospira* | *Leptospira kirschneri* | 1 | 1 |
| *Leptospira* | *Leptospira kmetyi* | 1 | 1 |
| *Leptospira* | *Leptospira mayottensis* | 1 | 0 |
| *Leptospira* | *Leptospira noguchii* | 1 | 1 |
| *Leptospira* | *Leptospira santarosai* | 1 | 1 |
| *Leptospira* | *Leptospira weilii* | 1 | 1 |
| *Listeria* | *Listeria monocytogenes* | 1 | 1 |
| *Malacoplasma* | *Malacoplasma penetrans* | 1 | 0 |
| *Metamycoplasma* | *Metamycoplasma hominis* | 1 | 0 |
| *Metamycoplasma* | *Metamycoplasma salivarium* | 1 | 0 |
| *Methylorubrum* | *Methylorubrum extorquens* | 1 | 1 |
| *Moraxella* | *Moraxella catarrhalis* | 1 | 1 |
| *Moraxella* | *Moraxella nonliquefaciens* | 1 | 1 |
| *Morganella* | *Morganella morganii* | 1 | 1 |
| *Mycobacterium* | *Mycobacterium avium* | 1 | 1 |
| *Mycobacterium* | *Mycobacterium intracellulare* | 1 | 1 |
| *Mycobacterium* | *Mycobacterium kansasii* | 1 | 1 |
| *Mycobacterium* | *Mycobacterium leprae* | 1 | 1 |
| *Mycobacterium* | *Mycobacterium lepromatosis* | 1 | 1 |
| *Mycobacterium* | *Mycobacterium malmoense* | 1 | 1 |
| *Mycobacterium* | *Mycobacterium marinum* | 1 | 1 |
| *Mycobacterium* | *Mycobacterium simiae* | 1 | 1 |
| *Mycobacterium* | *Mycobacterium tuberculosis* | 1 | 1 |
| *Mycobacterium* | *Mycobacterium ulcerans* | 0 | 1 |
| *Mycobacterium* | *Mycobacterium xenopi* | 0 | 1 |
| *Mycobacteroides* | *Mycobacteroides abscessus* | 1 | 1 |
| *Mycobacteroides* | *Mycobacteroides chelonae* | 1 | 1 |
| *Mycolicibacter* | *Mycolicibacter terrae* | 1 | 1 |
| *Mycolicibacterium* | *Mycolicibacterium fortuitum* | 1 | 1 |
| *Mycolicibacterium* | *Mycolicibacterium smegmatis* | 1 | 1 |
| *Mycoplasmoides* | *Mycoplasmoides genitalium* | 1 | 0 |
| *Mycoplasmoides* | *Mycoplasmoides pneumoniae* | 1 | 0 |
| *Mycoplasmopsis* | *Mycoplasmopsis caviae* | 1 | 0 |
| *Mycoplasmopsis* | *Mycoplasmopsis fermentans* | 1 | 1 |
| *Nakaseomyces* | *Nakaseomyces glabratus* | 1 | 1 |
| *Neisseria* | *Neisseria gonorrhoeae* | 1 | 1 |
| *Neisseria* | *Neisseria meningitidis* | 1 | 1 |
| *Neorickettsia* | *Neorickettsia sennetsu* | 1 | 0 |
| *Nocardia* | *Nocardia asteroides* | 1 | 1 |
| *Nocardia* | *Nocardia brasiliensis* | 1 | 1 |
| *Nocardia* | *Nocardia farcinica* | 1 | 1 |
| *Nocardia* | *Nocardia nova* | 1 | 1 |
| *Nocardia* | *Nocardia otitidiscaviarum* | 1 | 1 |
| *Orientia* | *Orientia tsutsugamushi* | 1 | 1 |
| *Paraclostridium* | *Paraclostridium bifermentans* | 1 | 1 |
| *Parvimonas* | *Parvimonas micra* | 1 | 1 |
| *Pasteurella* | *Pasteurella multocida* | 1 | 0 |
| *Photobacterium* | *Photobacterium damselae* | 1 | 1 |
| *Pichia* | *Pichia kudriavzevii* | 1 | 1 |
| *Plesiomonas* | *Plesiomonas shigelloides* | 1 | 1 |
| *Porphyromonas* | *Porphyromonas gingivalis* | 1 | 1 |
| *Prescottella* | *Prescottella equi* | 1 | 1 |
| *Prevotella* | *Prevotella bivia* | 1 | 1 |
| *Prevotella* | *Prevotella intermedia* | 1 | 1 |
| *Prevotella* | *Prevotella melaninogenica* | 1 | 1 |
| *Proteus* | *Proteus mirabilis* | 1 | 1 |
| *Proteus* | *Proteus penneri* | 1 | 0 |
| *Proteus* | *Proteus vulgaris* | 1 | 1 |
| *Providencia* | *Providencia alcalifaciens* | 1 | 1 |
| *Providencia* | *Providencia rettgeri* | 1 | 1 |
| *Pseudomonas* | *Pseudomonas aeruginosa* | 1 | 1 |
| *Pseudomonas* | *Pseudomonas fluorescens* | 1 | 1 |
| *Pseudomonas* | *Pseudomonas putida* | 1 | 1 |
| *Ralstonia* | *Ralstonia pickettii* | 1 | 1 |
| *Rickettsia* | *Rickettsia akari* | 1 | 0 |
| *Rickettsia* | *Rickettsia australis* | 1 | 0 |
| *Rickettsia* | *Rickettsia canadensis* | 1 | 0 |
| *Rickettsia* | *Rickettsia conorii* | 1 | 1 |
| *Rickettsia* | *Rickettsia helvetica* | 1 | 0 |
| *Rickettsia* | *Rickettsia prowazekii* | 1 | 0 |
| *Rickettsia* | *Rickettsia typhi* | 1 | 0 |
| *Rothia* | *Rothia dentocariosa* | 1 | 1 |
| *Saccharomyces* | *Saccharomyces cerevisiae* | 1 | 1 |
| *Salmonella* | *Salmonella bongori* | 1 | 1 |
| *Salmonella* | *Salmonella enterica* | 1 | 1 |
| *Serratia* | *Serratia marcescens* | 1 | 1 |
| *Shewanella* | *Shewanella algae* | 1 | 1 |
| *Shigella* | *Shigella boydii* | 1 | 1 |
| *Shigella* | *Shigella dysenteriae* | 1 | 1 |
| *Shigella* | *Shigella flexneri* | 1 | 1 |
| *Shigella* | *Shigella sonnei* | 0 | 1 |
| *Staphylococcus* | *Staphylococcus aureus* | 1 | 1 |
| *Staphylococcus* | *Staphylococcus capitis* | 1 | 1 |
| *Staphylococcus* | *Staphylococcus epidermidis* | 1 | 1 |
| *Staphylococcus* | *Staphylococcus haemolyticus* | 1 | 1 |
| *Staphylococcus* | *Staphylococcus lugdunensis* | 1 | 1 |
| *Staphylococcus* | *Staphylococcus pseudintermedius* | 1 | 1 |
| *Staphylococcus* | *Staphylococcus saccharolyticus* | 1 | 1 |
| *Staphylococcus* | *Staphylococcus saprophyticus* | 1 | 1 |
| *Stenotrophomonas* | *Stenotrophomonas maltophilia* | 1 | 1 |
| *Streptobacillus* | *Streptobacillus moniliformis* | 1 | 1 |
| *Streptococcus* | *Streptococcus agalactiae* | 1 | 1 |
| *Streptococcus* | *Streptococcus anginosus* | 1 | 1 |
| *Streptococcus* | *Streptococcus dysgalactiae* | 1 | 1 |
| *Streptococcus* | *Streptococcus equi* | 1 | 1 |
| *Streptococcus* | *Streptococcus equinus* | 1 | 1 |
| *Streptococcus* | *Streptococcus gordonii* | 1 | 1 |
| *Streptococcus* | *Streptococcus iniae* | 1 | 0 |
| *Streptococcus* | *Streptococcus mitis* | 1 | 1 |
| *Streptococcus* | *Streptococcus mutans* | 1 | 1 |
| *Streptococcus* | *Streptococcus oralis* | 1 | 1 |
| *Streptococcus* | *Streptococcus pneumoniae* | 1 | 1 |
| *Streptococcus* | *Streptococcus pyogenes* | 1 | 1 |
| *Streptococcus* | *Streptococcus sanguinis* | 1 | 1 |
| *Streptococcus* | *Streptococcus sobrinus* | 1 | 1 |
| *Streptococcus* | *Streptococcus suis* | 1 | 1 |
| *Talaromyces* | *Talaromyces marneffei* | 1 | 1 |
| *Tannerella* | *Tannerella forsythia* | 1 | 1 |
| *Treponema* | *Treponema denticola* | 1 | 1 |
| *Treponema* | *Treponema pallidum* | 1 | 0 |
| *Treponema* | *Treponema vincentii* | 1 | 1 |
| *Tropheryma* | *Tropheryma whipplei* | 0 | 1 |
| *Trueperella* | *Trueperella pyogenes* | 1 | 1 |
| *Ureaplasma* | *Ureaplasma parvum* | 1 | 1 |
| *Ureaplasma* | *Ureaplasma urealyticum* | 1 | 0 |
| *Vibrio* | *Vibrio alginolyticus* | 1 | 1 |
| *Vibrio* | *Vibrio cholerae* | 1 | 1 |
| *Vibrio* | *Vibrio parahaemolyticus* | 1 | 1 |
| *Vibrio* | *Vibrio vulnificus* | 1 | 1 |
| *Yersinia* | *Yersinia enterocolitica* | 1 | 1 |
| *Yersinia* | *Yersinia pestis* | 1 | 1 |
| *Yersinia* | *Yersinia pseudotuberculosis* | 1 | 0 |

**Table S4. Nucleic acid yield for each sample**

| Household id | Soil floor sample  ng/μL | Cow dung sample  ng/μL |
| --- | --- | --- |
| 1 | 34.9 | 237 |
| 2 | 36.5 | 186 |
| 3 | 32.8 | 228 |
| 4 | 36.5 | 208 |
| 5 | 43.1 | 298 |
| 6 | 38.9 | 320 |
| 7 | 29.7 | 212 |
| 8 | 39.3 | 237 |
| 9 | 37.3 | 197 |
| 10 | 35.2 | 282 |

##

##

##

##


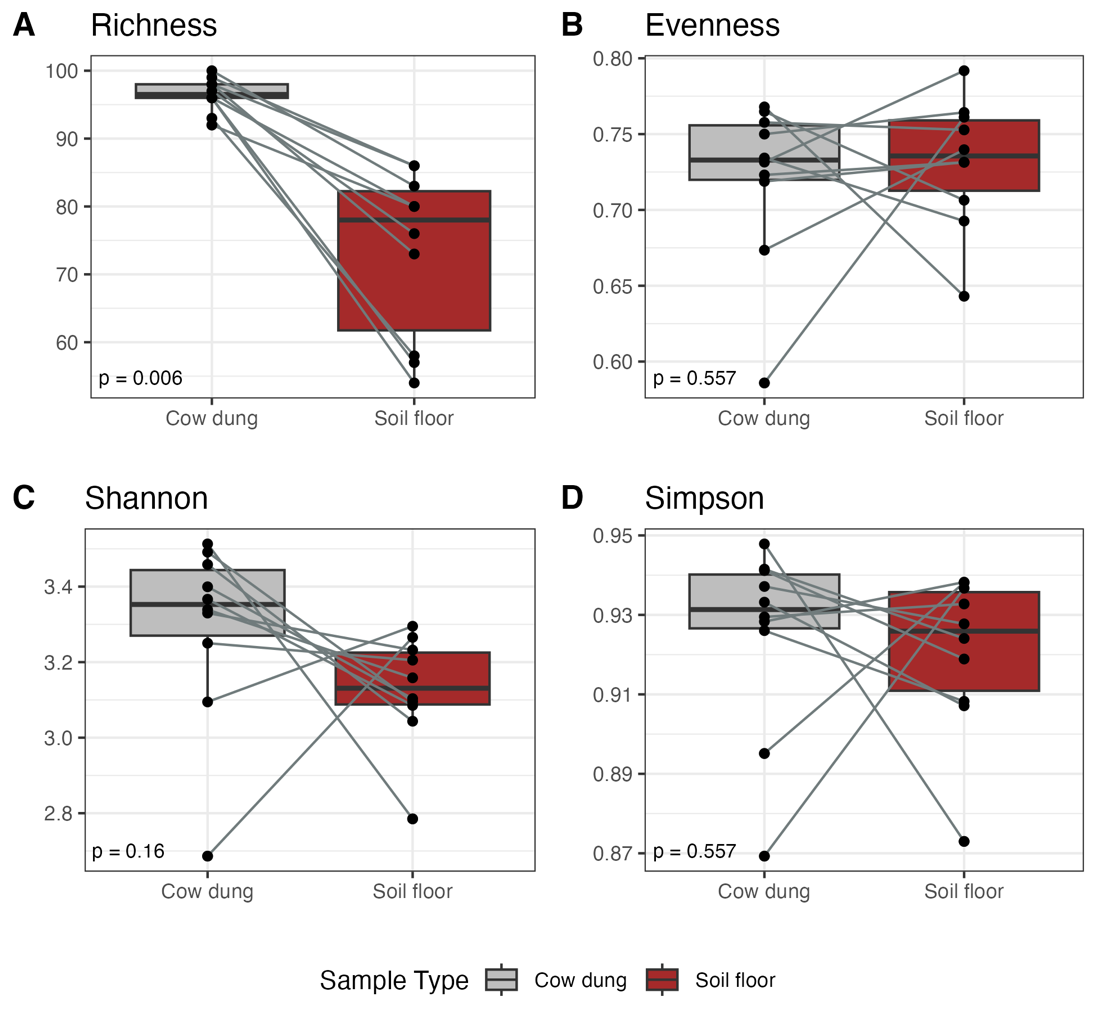


**Figure S1. Alpha-diversity indices for potential pathogen genera by sample type.** Includes 10 paired cow dung and soil floor samples. Indices were compared between sample types using the Wilcoxon signed-rank test.

##
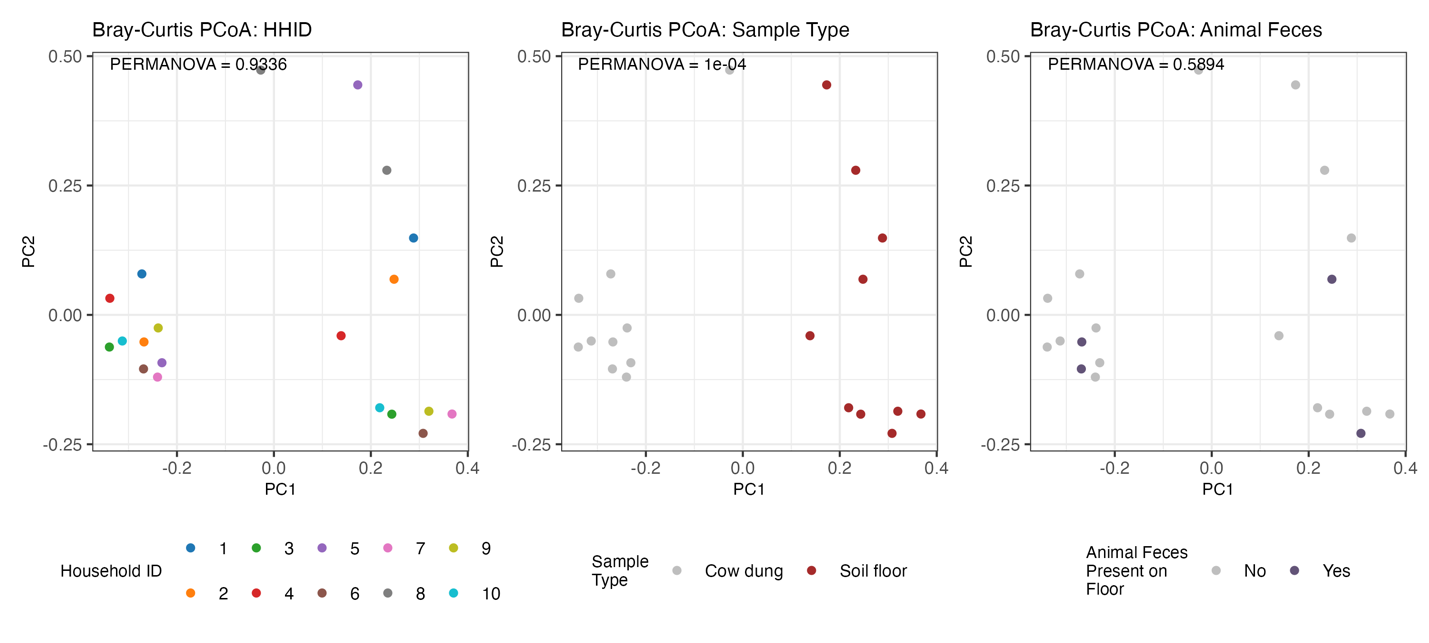


**Figure S2. Bray Curtis dissimilarity between communities of potential pathogen species by household membership, sample type, and presence of animal feces on the household floor.** Includes 10 household-paired cow dung and soil floor samples. Bray-Curtis dissimilarity was compared between sample types using PERMANOVA.

##

**Figure S3. Number of antibiotic resistance genes (ARGs) in each human health risk quartile in each sample.** Based on classifications in Zhang et al., 2022 (<https://doi.org/10.1038/s41467-022-29283-8>).

 **Figure S4. Heatmap of human health risk of antibiotic resistance genes detected in cow dung and soil samples.** Tile colors indicate human health risk classifications in Zhang et al., 2022 (<https://doi.org/10.1038/s41467-022-29283-8>). Includes genes with read coverage breadth > 10% and > 5 reads mapped or contig coverage breadth > 10%. Excludes ARGs that were not listed included in the analysis in Zhang et al., 2022.
